# Clinical characteristics and exploratory outcome associations among patients hospitalized with acute febrile illness during a post-flood period: a multicenter cohort study from Vietnam

**DOI:** 10.1590/S1678-9946202668068

**Published:** 2026-10-05

**Authors:** Tuan Quoc Le, Thuy Ngoc Tran, Ngoc Quynh Bui, Hoang Thi Thuy Kieu, Thi Thao Ma, Hoang Duc Dong

**Affiliations:** 1 Phu Tho Provincial Center for Disease Control, Phu Tho, Vietnam; 2 Thai Nguyen University of Medicine and Pharmacy, Faculty of Medical Technology, Thai Nguyen, Viet Nam; 3 Thai Nguyen University of Medicine and Pharmacy, Thai Nguyen, Viet Nam; 4 Thai Nguyen University of Medicine and Pharmacy, Department of Internal Medicine, Thai Nguyen, Viet Nam

**Keywords:** Post-flood period, Acute febrile illness, Environmental exposure, Procalcitonin, C-reactive protein.

## Abstract

Flooding can disrupt sanitation, contaminate water sources, and alter patterns of infectious disease; however, causal attribution of an individual febrile illness episode to flood exposure is difficult in retrospective hospital-based data. This study described hospitalized patients with acute febrile illness during a post-flood period in northern Vietnam, exploring associations with adverse short-term outcomes. This multicenter retrospective observational cohort included 237 consecutive patients admitted in Thai Nguyen province from October to November 2025. Demographic, documented environmental, clinical, laboratory, etiologic, and treatment data were obtained from medical records. Severe disease occurred in 8.4% and treatment non-response in 6.8% of patients. At admission, mean high-sensitivity C-reactive protein (hs-CRP) was 103.3 ± 98.3 mg/L, procalcitonin (PCT) was 6.7 ± 11.6 ng/mL, AST was 55.6 ± 61.4 U/L, ALT was 42.5 ± 46.2 U/L, and GGT was 79.9 ± 70.7 U/L. Recorded etiologic diagnoses most commonly included Escherichia coli (33.3%), Vibrio cholerae (18.1%), influenza viruses (13.5%), Leptospira species (11.0%), and Streptococcus pneumoniae (11.0%). Ceftriaxone was administered to 61.5% of patients. Separate univariable exploratory analyses associated severe-flood residence, non-clean water use, chronic disease history, hs-CRP > 100 mg/L, PCT > 2 ng/mL, and fever duration ≥ 3 days with adverse outcomes. These findings represent within-cohort associations among patients from flood-affected communities and do not indicate that flooding caused the illness episodes or outcomes. Most patients exhibited favorable short-term outcomes.

## INTRODUCTION

Floods can contaminate water supplies, disrupt sanitation and healthcare access, and increase contact with environmental hazards, with important health consequences in low- and middle-income settings^1^ ^-^ ^3^. Acute febrile illness after flooding may include enteric, respiratory, vector-borne, and zoonotic infections^4^ ^,^ ^5^. A key evidence gap is the limited hospital-based description of clinical, laboratory, etiologic, and treatment patterns during post-flood periods in northern Vietnam. Temporal proximity to flooding does not establish causation for an individual illness episode; retrospective cohorts are better suited to describe patterns and generate hypotheses than to estimate a flood-attributable effect.

Vietnam is vulnerable to recurrent storms and flooding. During the October-November 2025 study period, hospitals in Thai Nguyen served communities with documented temporary inundation and, in more severely affected areas, disruption of access and contamination of local water and sanitation systems. Quantitative hydrological measures for the event were not available in the medical records^6^ ^-^ ^8^.

High-sensitivity C-reactive protein (hs-CRP) and procalcitonin (PCT) are routinely available markers of systemic inflammation and infection severity^9^ ^-^ ^12^. Their role here was not to establish novel prognostic thresholds, but to examine whether routinely recorded markers revealed clinically relevant patterns in this post-flood hospital cohort.

Environmental documentation, comorbidity, delayed presentation, and inflammatory response may be interrelated. Accordingly, analyses should distinguish the post-flood context from individual-level causal attribution and treat observed associations as potentially confounded and exploratory^13^ ^-^ ^17^.

This study describes demographic, documented environmental, clinical, laboratory, recorded etiologic, and treatment characteristics of patients hospitalized with acute febrile illness during a post-flood period in Thai Nguyen province. Additionally, it explores univariable associations with short-term clinical outcomes. No causal effect or validated prediction model was intended.

### Ethics

Given the retrospective design and use of anonymized medical records, the requirement for informed consent was waived by the committee. Study execution complied with the Declaration of Helsinki.

## MATERIALS AND METHODS

### Study design and reporting guideline

This multicenter retrospective observational cohort study described hospitalized patients with acute febrile illness during a post-flood period in Thai Nguyen province, Vietnam, from October to November 2025. The study evaluated demographic characteristics, documented environmental indicators, clinical and admission laboratory findings, recorded etiologic diagnoses, antibiotic utilization, disease severity, and treatment response. Reporting followed the Strengthening the Reporting of Observational Studies in Epidemiology (STROBE) guidelines. Supplementary Table S1 provides a complete checklist.

#### Study setting

Patients were recruited consecutively from Thai Nguyen Central Hospital, Hospital A, Thai Nguyen, and Thai Nguyen University of Medicine and Pharmacy Hospital. These hospitals provide inpatient care to communities affected by seasonal flooding in northern Vietnam. The study followed a major local flooding event with heterogeneous documented impacts, ranging from temporary water coverage to extensive inundation with disruption of care access and water-sanitation systems. No unaffected comparison group or independent event-level hydrological dataset was available.

#### Participants

#### Inclusion criteria

Patients aged ≥ 16 years; admitted with acute febrile illness, defined as a body temperature ≥ 38 °C; residing in an area documented as affected by the recent flood or with documented direct or indirect floodwater contact; and who had the demographic, environmental, clinical, and laboratory variables required for the planned analyses were eligible. These criteria defined a post-flood hospital cohort and did not establish that flooding caused the febrile illness.

#### Exclusion criteria

Patients with incomplete records for the required analytic variables, fever clearly attributable to a non-infectious cause, or readmitted for the same illness episode during the study period were excluded. The final analytic cohort comprised 237 patients. The exact number initially screened, and the number excluded for each reason could not be reconstructed from the de-identified analytic files, which is acknowledged as a limitation.

#### Data sources and variable definitions

Data were extracted retrospectively from electronic medical records using a standardized case report form. Variables corresponded to the demographic, environmental, clinical, laboratory, etiologic, and treatment data summarized in Tables 1 to 4 and Figures 1 and 2. No missing values were imputed. All analyses used the complete data available for the final eligible cohort.

#### Demographic and environmental variables

Demographic variables included sex, age, occupation, and treatment hospital. Age was grouped as < 20, 20-39, 40-59, and ≥ 60 years for descriptive analyses. Environmental indicators were obtained from medical-record documentation rather than independently verified geospatial or hydrological measurements. Residence was categorized as a mild-flood area when records described temporary water coverage without major disruption to care access or to essential services, and as a severely flood-affected area when records described extensive inundation with major disruption or contamination of local water and sanitation systems. Water used during and after flooding was categorized as clean when obtained from treated or protected sources-including piped water, bottled water, or protected wells-and as non-clean when obtained from floodwater, surface water, unprotected wells, visibly contaminated water, or another source documented as unsafe.

#### Clinical variables

Clinical variables included maximum body temperature recorded at admission, fever duration before admission, treatment duration, chronic disease history, disease severity, and treatment response. Chronic disease history was defined as a physician-diagnosed condition documented before admission. Recorded conditions included cirrhosis, hyperthyroidism, hypertension, hepatitis B, drug addiction, pneumonia, and diabetes. Fever duration was measured as the number of days from symptom onset to hospital admission.

#### Laboratory variables

All laboratory values reported in Table 2 represent the first available measurements obtained at hospital admission. Parameters included white blood cell count, neutrophil and lymphocyte percentages, red blood cell count, hemoglobin, platelet count, international normalized ratio, glucose, urea, creatinine, aspartate aminotransferase, alanine aminotransferase, gamma-glutamyl transferase, sodium, potassium, chloride, hs-CRP, and PCT. For exploratory regression analyses, hs-CRP was categorized as ≤ 100 or > 100 mg/L and PCT as ≤ 2 or > 2 ng/mL.

Etiologic diagnoses were obtained retrospectively from diagnostic results and clinician documentation during the index hospitalization, from admission to discharge. Testing was ordered according to hospital protocols and the treating physician’s clinical judgment rather than a uniform study-specific diagnostic panel. Depending on the suspected syndrome, specimens included blood, urine, stool, sputum, respiratory samples, and other clinically indicated samples.

Bacterial organisms were recorded from culture-based or other routine hospital microbiology results. Respiratory viral infections, including influenza, were recorded when supported by antigen-based or molecular tests documented in the record. Dengue virus, hepatitis A virus, Leptospira species, and Rickettsia species were recorded when supported by serologic, antigen-based, or molecular testing according to local protocols.

A recorded etiologic diagnosis was accepted when the treating physician documented an organism as clinically relevant and compatible with the syndrome, specimen source, and clinical course. Since confirmation methods differed by pathogen and hospital and were not consistently coded in the de-identified files, recorded etiologic diagnoses could not be reliably separated into uniformly laboratory-confirmed and clinically assigned categories.

#### Microbiological investigations and antibiotic use

Figure 1 descriptively summarizes the recorded etiologic diagnoses for the 237 patients. These categories were obtained from the medical records and should not be

**Figure 1:**
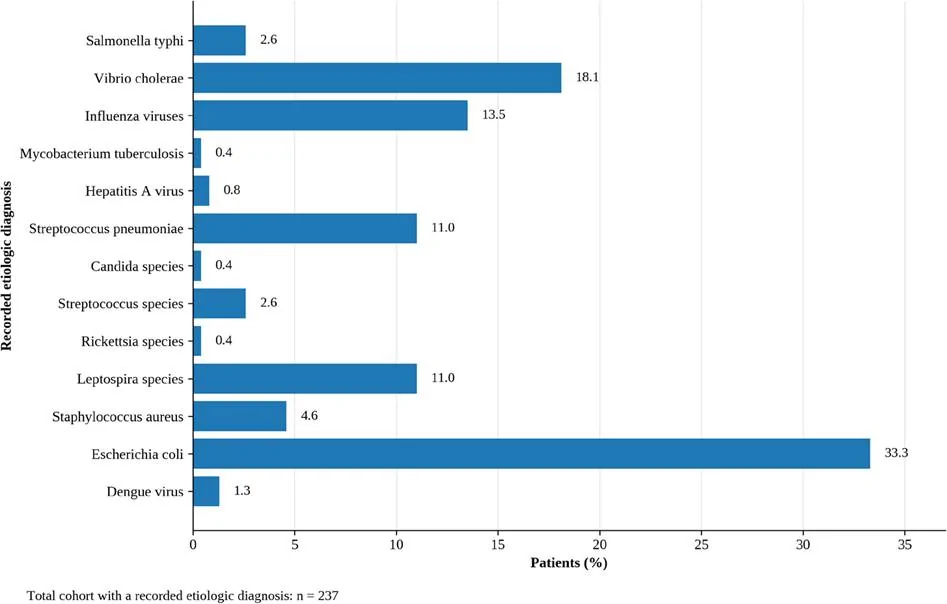
Distribution of recorded infectious etiologic diagnoses among 237 patients hospitalized with acute febrile illness during the post-flood period. Values presented as percentages. Diagnoses were obtained from medical records and were not assigned using a uniform study-specific diagnostic panel; the distribution should not be interpreted as the prevalence of uniformly laboratory-confirmed infections.

interpreted as prevalence estimates of uniformly laboratory-confirmed infection. Recorded diagnoses included dengue virus, Escherichia coli, Staphylococcus aureus, Leptospira species, Rickettsia species, Streptococcus species, Candida species, Streptococcus pneumoniae, hepatitis A virus, Mycobacterium tuberculosis, influenza viruses, Vibrio cholerae, and Salmonella typhi. Antibiotics administered at any time during the index hospitalization are summarized in Figure 2.

**Figure 2:**
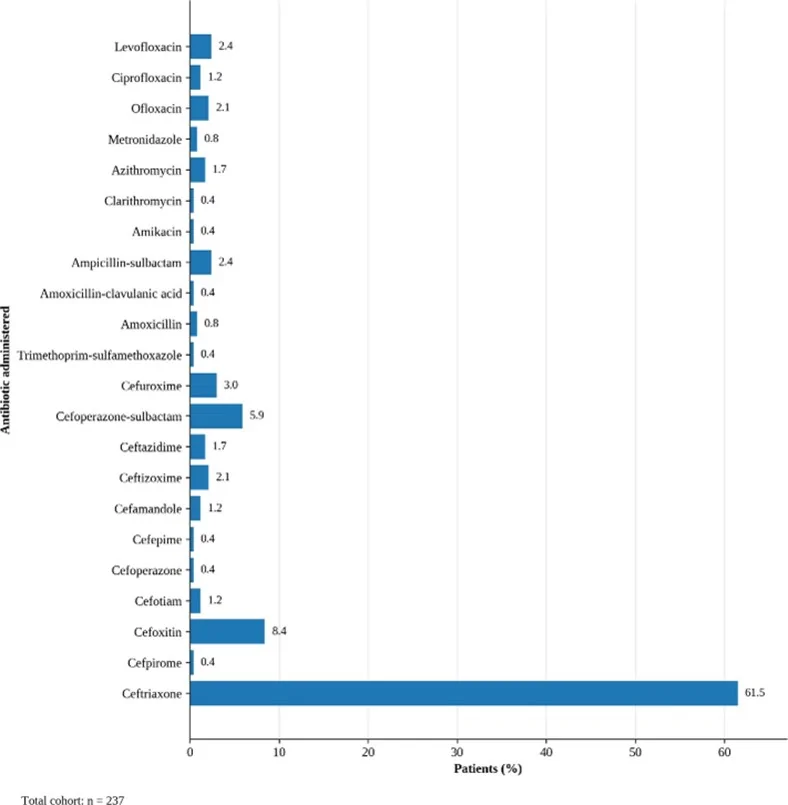
Distribution of antibiotics administered during the index hospitalization among 237 patients with acute febrile illness during the post-flood period. Values presented as percentages; percentages may not sum to 100 due to rounding.

### Outcomes

#### Primary outcome: disease severity

Disease severity constituted the primary outcome, categorized as mild to moderate or severe. Severe disease was defined by the presence of at least one of the following: requirement for intensive monitoring or intensive care unit admission; hemodynamic instability requiring vasopressor support; organ dysfunction, including acute kidney injury, hepatic dysfunction with an international normalized ratio > 1.5, or respiratory compromise requiring oxygen support; or a documented clinical diagnosis of sepsis or septic shock. All other cases were classified as mild to moderate.

#### Secondary outcome: treatment response

Secondary outcome consisted of treatment response, categorized as response or non-response. Treatment response was defined as clinical improvement with resolution of fever and major symptoms, no requirement for escalation of antimicrobial therapy, and discharge without complications. Non-response was defined as persistent fever beyond 72 hours despite treatment, clinical deterioration, escalation to broader-spectrum antimicrobial therapy, or transfer to an intensive care unit.

#### Bias control

Despite use of consecutive inclusion and a standardized case report form to reduce selection and extraction bias, selection bias remained because eligibility required sufficiently complete records. Environmental classifications depended on medical-record documentation, the cohort had no unaffected comparison group, and diagnostic testing was indication-driven. These features limit causal interpretation and may produce differential etiologic ascertainment.

### Statistical analysis

Continuous variables were summarized using minimum, maximum, mean, and standard deviation values, as appropriate; categorical variables were summarized as frequencies and percentages. Separate univariable logistic regression models examined associations with severe disease and treatment non-response, with odds ratios (ORs), 95% confidence intervals (CIs), and two-sided p-values. Variables were categorized as follows: sex (female; male); age (< 50; ≥ 50 years); residence (mild-flood; severely flood-affected area); water use during and after flooding (clean; non-clean); chronic disease history (no; yes); body temperature (< 39; ≥ 39 °C); hs-CRP (≤ 100; > 100 mg/L); PCT (≤ 2; > 2 ng/mL); and fever duration (< 3; ≥ 3 days). The study sample was a census of consecutive eligible admissions during the fixed study period; no a priori power calculation was performed. Due to only 20 severe events and 16 non-response events recorded, multivariable modeling was not performed. No adjustment for multiple comparisons was applied. All p-values were treated as descriptive, and interpretation emphasized effect sizes, confidence intervals, and consistency rather than dichotomous significance. These analyses were hypothesis-generating and vulnerable to sparse-data instability and confounding. SPSS Statistics (version 22.0) was used.

## RESULTS

### Baseline characteristics

Our analysis included a total of 237 patients hospitalized with acute febrile illness during the post-flood period. Most patients were male (65.0%) and were aged between 40-59 years (39.2%), followed by 20-39 years (34.2%). Most frequent occupational category was freelance work (28.7%). Most patients were treated at the Thai Nguyen Central Hospital (85.2%).

Regarding documented environmental indicators, 157 patients (66.2%) resided in mild-flood areas and 80 (33.8%) in severely flood-affected areas. During flooding, 94 patients (39.7%) were classified as using non-clean water; after flooding, 89 (37.6%) were classified as using non-clean water. Maximum admission temperature was ≥ 39 °C in 120 patients (50.6%). Most participants had no documented chronic disease (93.2%). Overall, 20 cases (8.4%) were classified as severe and 217 (91.6%) as mild to moderate (Table 1).

### Treatment characteristics and outcomes

Mean treatment duration was 5.1 ± 3.2 days, and mean fever duration was 2.1 ± 1.9 days. Most patients responded to treatment (93.2%); only 6.8% did not respond (Table 1).

**Table 1:** Baseline demographic and clinical characteristics of acute febrile illness during the post-flood period.

Patient characteristics	Min	Max	Mean ± SD or n (%)
Sex, n (%)			
Male			154 (65.0)
Female			83 (35.0)
Age group, n (%)			
< 20			23 (9.7)
20-39			81 (34.2)
40-59			93 (39.2)
≥ 60			40 (16.9)
Occupation, n (%)			
Farmer			32 (13.5)
Worker			38 (16.0)
Student			34 (14.3)
Officials			51 (21.5)
Freelance			68 (28.7)
Other			14 (5.9)
Treatment hospital, n (%)			
Thai Nguyen Central Hospital			202 (85.2)
Hospital A, Thai Nguyen			11 (4.6)
Nguyen University of medicine and Pharmacy Hospital			24 (10.1)
Place of residence, n (%)			
Mild-flood areas			157 (66.2)
Severely flood-affected areas			80 (33.8)
Water source during flooding, n (%)			
Clean water			143 (60.3)
Non-clean water			94 (39.7)
Water source after flooding, n (%)			
Clan water			148 (62.4)
Non-clean water			89 (37.6)
Maximum body temperature n (%)			
< 39°C			117 (49.4)
≥ 39°C			120 (50.6)
Chronic disease history, n (%)			
No chronic disease			221 (93.2)
Cirrhosis			2 (0.8)
Hyperthyroidism			2 (0.8)
Hypertension			4 (1.7)
Hepatitis B			3 (1.3)
Drug addiction			1 (0.4)
Pneumonia			1 (0.4)
Diabetes			3 (1.3)
Disease severity, n (%)			
Mild to moderate			217 (91.6)
Severe			20 (8.4)
Treatment duration (days)	1	20	5.1 ± 3.2
Fever duration (days)	1	14	2.1 ± 1.9
Treatment response, n (%)			
Non-response			16 (6.8)
Response			221 (93.2)

### Laboratory findings

At admission, mean white blood cell count was 9.4 ± 4.2 ×109/L. Mean neutrophil percentage was 79.5 ± 15.1%, and mean lymphocyte percentage was 12.1 ± 10.7%. Mean hemoglobin level was 134.5 ± 17.3 g/L, and mean platelet count was 214.7 ± 82.2 ×109/L.

All values in Table 2 were measured at hospital admission. Mean hs-CRP was 103.3 ± 98.3 mg/L and mean PCT was 6.7 ± 11.6 ng/mL. Mean AST, ALT, and GGT were 55.6 ± 61.4 U/L, 42.5 ± 46.2 U/L, and 79.9 ± 70.7 U/L, respectively. Mean creatinine was 103.1 ± 65.4 µmol/L and mean INR was 1.2 ± 0.1.

**Table 2:** Laboratory findings at admission.

Laboratory parameters	Min	Max	Mean ± SD
WBC (109/L)	2	40	9.4 ± 4.2
NEU (%)	7.6	96.6	79.5 ± 15.1
LYM (%)	2	69	12.1 ± 10.7
RBC (1012/L)	2	6	4.6 ± 0.6
Hb (g/L)	66	170	134.5 ± 17.3
PLT (109/L)	13	577	214.7 ± 82.2
INR	1.0	1.4	1.2 ± 0.1
Glucose (mmol/L)	3	25	6.9 ± 2.6
Urea (mmol/L)	1	26	5.6 ± 2.8
Creatinine (µmol/L)	26	797	103.1 ± 65.4
AST (U/L)	4	398	55.6 ± 61.4
ALT (U/L)	3.5	272.4	42.5 ± 46.2
GGT (U/L)	9	295	79.9 ± 70.7
Sodium (mmol/L)	126	143	135.9 ± 3.0
Potassium (mmol/L)	3	6	3.7 ± 0.6
Chloride (mmol/L)	91	121	100.6 ± 3.7
hs-CRP (mg/L)	1	386	103.3 ± 98.3
PCT (ng/mL)	1	44.25	6.7 ± 11.6

All laboratory values were obtained at hospital admission; WBC = white blood cell count; NEU = neutrophils; LYM = lymphocytes; RBC = red blood cell count; Hb = hemoglobin; PLT = platelets; INR = international normalized ratio; ALT = alanine aminotransferase; AST = aspartate aminotransferase; GGT = gamma-glutamyl transferase; hs-CRP = high-sensitivity C-reactive protein; PCT = procalcitonin.

### Recorded infectious etiologies

Recorded etiologic diagnosis was available for all 237 patients. Escherichia coli accounted for 33.3% of recorded diagnoses, followed by Vibrio cholerae (18.1%), influenza viruses (13.5%), Leptospira species (11.0%), and Streptococcus pneumoniae (11.0%). Figure 1 shows the less frequent categories. Since testing was not standardized and the de-identified files did not consistently distinguish laboratory-confirmed from clinically assigned diagnoses, these percentages are descriptive categories from the medical record and should not be interpreted as pathogen prevalence estimates.

### Antibiotic utilization patterns

Ceftriaxone was the most frequently administered antibiotic (61.5%), followed by cefoxitin (8.4%), cefoperazone-sulbactam (5.9%), and cefuroxime (3.0%). Figure 2 shows the distribution of antibiotics administered to patients with acute febrile illness during the post-flood period.

### Exploratory associations with disease severity

Separate univariable analyses associated severe-flood residence with severe disease (OR 3.792, 95% CI 1.079-13.330; p = 0.027). Non-clean water use during flooding (OR 3.118, 95% CI 1.195-8.137; p = 0.015) and after flooding (OR 2.727, 95% CI 1.069-6.960; p = 0.030), chronic disease history (OR 8.059, 95% CI 2.983-21.771; p = 0.001), hs-CRP > 100 mg/L (OR 3.586, 95% CI 1.399-9.189; p = 0.005), PCT > 2 ng/mL (OR 5.623, 95% CI 2.082-15.187; p = 0.001), and fever duration ≥ 3 days (OR 7.923, 95% CI 3.022-20.773; p = 0.001) also correlated with severe disease. Male sex had an imprecise borderline estimate (OR 3.309, 95% CI 0.941-11.642; p = 0.050), whereas age and body temperature showed no association. These unadjusted estimates are exploratory, were not corrected for multiple testing, and should not be interpreted as independent predictors (Table 3).

**Table 3:** Exploratory univariable associations with disease severity.

Variables	Odds ratio (OR)	95% CI	p
Sex			
Female			
Male	3309	0.941-11.642	0.050
Age			
< 50			
≥ 50	2014	0.802-5.058	0.130
Place of residence			
Mild-flood areas			
Severely flood-affected areas	3792	1.079-13.330	0.027
Water use during the flooding period			
Clean water			
Non-clean water	3118	1.195-8.137	0.015
Water use after the flooding period			
Clean water			
Non-clean water	2727	1.069-6.960	0.030
Chronic disease history			
No			
Yes	8059	2.983-21.771	0.001
Body temperature (°C)			
< 39			
≥ 39	1909	0.734-4.969	0.179
hs-CRP (mg/L)			
≤ 100			
> 100	3586	1.399-9.189	0.005
PCT (ng/mL)			
≤ 2			
> 2	5623	2.082-15.187	0.001
Fever duration (days)			
< 3			
≥ 3	7923	3.022-20.773	0.001

Reference category is listed first; ORs are from separate univariable logistic regression models; No adjustment for multiple comparisons was applied; estimates are exploratory and do not represent independent effects; CI = confidence interval; hs-CRP = high-sensitivity C-reactive protein; OR = odds ratio; PCT = procalcitonin.

### Exploratory associations with treatment response

Separate univariable analyses associated treatment non-response with severe-flood residence (OR 4.632, 95% CI 1.027-20.880; p = 0.030), non-clean water use during flooding (OR 3.658, 95% CI 1.228-10.897; p = 0.014) and after flooding (OR 2.996, 95% CI 1.050-8.551; p = 0.033), chronic disease history (OR 7.035, 95% CI 2.386-20.747; p = 0 .001), hs-CRP > 100 mg/L ( OR 5.280, 95% CI 1.765-15.799; p = 0.001), PCT > 2 ng/mL (OR 8.609, 95% CI 2.950-25.120; p = 0.001), and fever duration ≥ 3 days (OR 3.300, 95% CI 1.125-9.679; p = 0.023). Sex, age, and body temperature showed no association. The small number of non-response events, wide CIs, lack of multiplicity correction, and absence of multivariable adjustment make these findings hypothesis-generating (Table 4).

**Table 4:** Exploratory univariable associations with treatment non-response.

Variables	Odds ratio (OR)	95% CI	p
Sex			
Female			
Male	1669	0.521-5.348	0.384
Age			
< 50			
≥ 50	1986	0.717-5.504	0.180
Place of residence			
Mild-flood areas			
Severely flood-affected areas	4632	1.027-20.880	0.030
Water use during the flooding period			
Clean water			
Non-clean water	3658	1.228-10.897	0.014
Water use after the flooding period			
Clean water			
Non-clean water	2996	1.050-8.551	0.033
Chronic disease history			
No			
Yes	7035	2.386-20.747	0.001
Body temperature (°C)			
< 39			
≥ 39	1682	0.591-4.786	0.326
hs-CRP (mg/L)			
≤ 100			
> 100	5280	1.765-15.799	0.001
PCT (ng/mL)			
≤ 2			
> 2	8609	2.950-25.120	0.001
Fever duration (days)			
< 3			
≥ 3	3300	1.125-9.679	0.023

Reference category is listed first; ORs are from separate univariable logistic regression models; No adjustment for multiple comparisons was applied; estimates are exploratory and do not represent independent effects; CI = confidence interval; hs-CRP = high-sensitivity C-reactive protein; OR = odds ratio; PCT = procalcitonin.

## DISCUSSION

### Environmental exposure and infectious spectrum

Within this flood-affected cohort, residence in a more severely affected area and documented non-clean water use were associated with adverse short-term outcomes. These internal exposure-gradient comparisons are not flood-versus-no-flood estimates and may reflect differences in comorbidity, healthcare access, illness severity at presentation, or other unmeasured factors. These findings therefore do not indicate that flood exposure caused the outcomes, despite correlating with established pathways by which contaminated water and disrupted sanitation can increase infectious hazards^18^ ^-^ ^21^. The heterogeneous recorded etiologic spectrum included enteric, respiratory, and zoonotic categories. However, test selection depended on clinical judgment, methods varied across hospitals and pathogens, and the records did not consistently distinguish laboratory-confirmed from clinically assigned diagnoses. The distribution presented in Figure 1 should therefore be interpreted as descriptive documentation within this hospital cohort rather than the true prevalence of etiologies after flooding ^22^ ^,^ ^23^.

### Inflammatory biomarkers and disease severity

Mean PCT was 6.7 ± 11.6 ng/mL; PCT > 2 ng/mL was associated with severe disease and treatment non-response in univariable analyses. This threshold should not be used as a stand-alone diagnostic criterion, and the wide variability and limited event counts require cautious interpretation ^24^ ^-^ ^27^. Mean hs-CRP was 103.3 ± 98.3 mg/L; hs-CRP > 100 mg/L was associated with both outcomes. These results are consistent with greater systemic inflammation among patients with worse short-term courses, but they do not suggest that hs-CRP or PCT independently determined progression. Larger prospective studies with prespecified adjustment are needed to evaluate incremental clinical utility ^28^ ^,^ ^29^.

### Gastrointestinal and hepatic involvement

Mean AST, ALT, and GGT values were above typical reference ranges, suggesting the occurrence of biochemical liver abnormalities in some patients. Several infections represented in the cohort can involve the liver, but the study did not include systematic bilirubin measurements, imaging, adjudicated hepatic diagnoses, or longitudinal hepatic outcomes ^30^ ^-^ ^33^.

Mean INR was 1.2 ± 0.1, which does not suggest frequent marked coagulopathy at cohort level. As only summary laboratory values were available, the data cannot establish the frequency, cause, or clinical consequences of hepatic involvement ^34^.

Accordingly, the hepatic findings are descriptive and support routine clinical assessment when indicated, rather than a specific gastroenterological conclusion.

### Comorbidities and host vulnerability

Documented chronic disease was associated with severe disease and non-response in univariable analyses. This is biologically plausible, but sparse cells and the lack of multivariable adjustment mean that the magnitude of association may be influenced by age, baseline frailty, type of infection, or other confounders ^35^ ^,^ ^36^.

### Fever duration and clinical progression

Fever duration ≥ 3 days was associated with both outcomes. Prolonged fever may reflect delayed presentation, a more persistent infection, or other clinical differences. The retrospective design cannot distinguish these explanations or establish that earlier treatment would have changed outcomes ^37^. Most patients exhibited favorable short-term outcomes. Ceftriaxone was the most frequently recorded antibiotic (61.5%) but indication, timing, microbiological appropriateness, and therapy changes were not evaluated. Its utilization pattern should therefore be interpreted descriptively and not as evidence of comparative effectiveness ^38^.

### Sex differences

Male sex presented a borderline association with severe disease, but the CI included 1.0 and the estimate was based on few events. Possible behavioral or occupational explanations remain speculative and require prospective evaluation ^39^.

### Clinical and public health implications

These findings support prospective evaluation of documented exposure indicators and admission biomarkers as components of post-flood triage. Such studies should use standardized exposure measures and diagnostic testing, prespecified multivariable models, and external validation. In the present cohort, water-source documentation and admission laboratory assessment were clinically informative descriptors but not validated prediction tools. At the public-health level, access to drinking water, sanitation, vaccination where appropriate, and vector and rodent control remain established priorities after flooding^40^. These general measures are supported by broader evidence rather than by causal estimates from this cohort.

### Limitations

This study has several limitations. Its retrospective hospital-based design and requirement for complete analytic records may have introduced selection bias as the exact number of patients screened and excluded could not be reconstructed. All participants came from flood-affected communities, thus the environmental analyses were internal comparisons without a flood-naive baseline. Exposure classifications were based on medical-record documentation rather than independent objective measurements. Diagnostic testing was indication-driven, heterogeneous, and not uniformly coded as laboratory-confirmed, creating differential ascertainment and limiting the interpretation of Figure 1. Study sample was a fixed-period census without an a priori power calculation. Only 20 severe events and 16 non-response events occurred. As 20 univariable tests were performed without multiplicity correction and no multivariable analysis was undertaken, this study’s estimates may be unstable, confounded, and vulnerable to chance findings. Bilirubin, adjudicated hepatic outcomes, and long-term outcomes were unavailable. Finally, findings from Thai Nguyen may not generalize to settings with different populations, flood characteristics, diagnostic resources, or pathogen profiles.

## CONCLUSIONS

Most patients hospitalized with acute febrile illness during a post-flood period in northern Vietnam presented favorable short-term outcomes. Documented environmental indicators, chronic comorbidity, prolonged ever, hs-CRP, and PCT showed exploratory univariable associations with adverse outcomes in this cohort. These findings are descriptive and hypothesis-generating; they do not establish flood causation or independent prognostic effects.

## Supplementary Material

Supplementary information are available at https://doi.org/10.48331/SCIELODATA.FJ0D1Q

Supplementary PDF

## Data Availability

The complete anonymized dataset supporting the findings of this study is available from https://doi.org/10.48331/SCIELODATA.FJ0D1Q
