# Supplementary material for: Clinical characteristics and exploratory outcome associations among patients hospitalized with acute febrile illness during a post-flood period: a multicenter cohort study from Vietnam
Source: Rev Inst Med Trop Sao Paulo. 2026 Oct 5;68:e68. doi: 10.1590/S1678-9946202668068 (PMC13641769; doi:10.1590/S1678-9946202668068)
Supplement: Supplementary file 1 [file 1678-9946-rimtsp-68-S1678-9946202668068-md1.pdf]

REVISTA DO INSTITUTO DE MEDICINA TROPICAL DE SÃO PAULO

JOURNAL OF THE SÃO PAULO INSTITUTE OF TROPICAL MEDICINE

SUPPLEMENTARY MATERIAL

https://doi.org/10.48331/SCIELODATA.FJ0D1Q

Clinical characteristics and exploratory outcome associations among patients hospitalized with acute febrile illness during a post-flood period: a multicenter cohort study from Vietnam

Tuan Quoc Le[1], Thuy Ngoc Tran[2], Ngoc Quynh Bui[3], Hoang Thi Thuy Kieu[3], Thi Thao Ma[3], Hoang Duc Dong[4]

[1]Phu Tho Provincial Center for Disease Control, Phu Tho, Vietnam

[2]Thai Nguyen University of Medicine and Pharmacy, Faculty of Medical Technology, Thai Nguyen, Viet Nam

[3]Thai Nguyen University of Medicine and Pharmacy, Thai Nguyen, Viet Nam

[4]Thai Nguyen University of Medicine and Pharmacy, Department of Internal Medicine, Thai Nguyen, Viet Nam

Correspondence to: Hoang Duc Dong
Thai Nguyen University of Medicine and Pharmacy, Department of Internal Medicine, 284 Luong Ngoc Quyen Street, Phan Dinh Phung Ward, Thai Nguyen Province, Vietnam
Tel: +84 985 123 432
E-mail: drhoang85@gmail.com

Received: 10 June 2026
Accepted: 20 August 2026

Editor: Thelma Suely Okay

Supplementary Table S1 - STROBE checklist.

Item	Recommendation	Reported information	Location
1	Title and abstract	State the study design and provide an informative, balanced summary.	Title; Abstract
2	Background/ rationale	Explain scientific background and rationale.	Introduction
3	Objectives	State specific objectives and prespecified hypotheses.	End of Introduction
4	Study design	Present key elements of study design early.	Methods - Study design
5	Setting	Describe setting, locations, and relevant dates.	Methods - Study setting
6	Participants	Give eligibility criteria, sources, and methods of selection.	Methods - Participants
7	Variables	Define outcomes, exposures, predictors, and confounders.	Methods - Variable definitions and Outcomes
8	Data sources/ measurement	Describe sources and assessment methods.	Methods - Data sources; Laboratory; Microbiology
9	Bias	Describe efforts to address potential bias.	Methods - Bias control; Limitations
10	Study size	Explain how study size was determined.	Methods - Statistical analysis
11	Quantitative variables	Explain handling and grouping of quantitative variables.	Methods - Statistical analysis
12	Statistical methods	Describe analyses, confounding approach, missing data, sensitivity/ subgroup methods.	Methods - Statistical analysis
13	Participants	Report numbers at stages and reasons for non-participation.	Methods - Exclusion criteria; Limitations
14	Descriptive data	Report participant characteristics and missingness.	Results; Tables 1–2
15	Outcome data	Report numbers of outcome events.	Results; Table 1
16	Main results	Give unadjusted estimates, precision, and category boundaries.	Results; Tables 3–4
17	Other analyses	Report other analyses performed.	Not applicable beyond descriptive analyses
18	Key results	Summarize key results with reference to objectives.	Discussion
19	Limitations	Discuss limitations, bias, and imprecision.	Discussion - Limitations
20	Interpretation	Give cautious overall interpretation.	Discussion; Conclusions
21	Generalisability	Discuss external validity.	Discussion - Limitations
22	Funding	No funding.	Author to confirm/add funding statement

This is an open-access article distributed under the terms of the Creative Commons Attribution License.
